# Ataxia telangiectasia and Rad3-related (ATR) inhibitor camonsertib dose optimization in patients with biomarker-selected advanced solid tumors (TRESR study)

**DOI:** 10.1093/jnci/djae098

**Published:** 2024-05-06

**Authors:** Elisa Fontana, Ezra Rosen, Elizabeth K Lee, Martin Højgaard, Niharika B Mettu, Stephanie Lheureux, Benedito A Carneiro, Gregory M Cote, Louise Carter, Ruth Plummer, Devalingam Mahalingam, Adrian J Fretland, Joseph D Schonhoft, Ian M Silverman, Marisa Wainszelbaum, Yi Xu, Danielle Ulanet, Maria Koehler, Timothy A Yap

**Affiliations:** Sarah Cannon Research Institute UK, London, UK; Early Drug Development and Breast Medicine Services, Division of Solid Tumor Oncology, Department of Medicine, Memorial Sloan Kettering Cancer Center, New York, NY, USA; Medical Oncology, Dana-Farber Cancer Institute, Boston, MA, USA; Department of Oncology, Rigshospitalet, Copenhagen, Denmark; Medical Oncology, Duke University, Durham, NC, USA; Princess Margaret Cancer Centre, Toronto, ON, Canada; Legorreta Cancer Center at Brown University and Lifespan Cancer Institute, Division of Hematology/Oncology, Department of Medicine, The Warren Alpert Medical School, Brown University, Providence, RI, USA; Mass General Cancer Center, Boston, MA, USA; Division of Cancer Sciences, The University of Manchester, Manchester, UK; The Christie NHS Foundation Trust, Manchester, UK; Sir Bobby Robson Cancer Trials Research Centre, Freeman Hospital, Newcastle upon Tyne, UK; Robert H. Lurie Comprehensive Cancer Center, Division of Hematology/Oncology, Northwestern University Feinberg School of Medicine, Chicago, IL, USA; Repare Therapeutics, Cambridge, MA, USA; Repare Therapeutics, Cambridge, MA, USA; Repare Therapeutics, Cambridge, MA, USA; Repare Therapeutics, Cambridge, MA, USA; Repare Therapeutics, Cambridge, MA, USA; Repare Therapeutics, Cambridge, MA, USA; Repare Therapeutics, Cambridge, MA, USA; Investigational Cancer Therapeutics, The University of Texas MD Anderson Cancer Center, Houston, TX, USA

## Abstract

**Background:**

Camonsertib is a selective oral inhibitor of ataxia telangiectasia and Rad3-related (ATR) kinase with demonstrated efficacy in tumors with DNA damage response gene deficiencies. On-target anemia is the main drug-related toxicity typically manifesting after the period of dose-limiting toxicity evaluation. Thus, dose and schedule optimization requires extended follow-up to assess prolonged treatment effects.

**Methods:**

Long-term safety, tolerability, and antitumor efficacy of 3 camonsertib monotherapy dosing regimens were assessed in the TRESR study dose-optimization phase: 160 mg once daily (QD) 3 days on, 4 days off (160 3/4; the preliminary recommended Phase II dose [RP2D]) and two step-down groups of 120 mg QD 3/4 (120 3/4) and 160 mg QD 3/4, 2 weeks on, 1 week off (160 3/4, 2/1w). Safety endpoints included incidence of treatment-related adverse events (TRAEs), dose modifications, and transfusions. Efficacy endpoints included overall response rate, clinical benefit rate, progression-free survival, and circulating tumor DNA (ctDNA)-based molecular response rate.

**Results:**

The analysis included 119 patients: 160 3/4 (n = 67), 120 3/4 (n = 25), and 160 3/4, 2/1w (n = 27) treated up to 117.1 weeks as of the data cutoff. The risk of developing grade 3 anemia was significantly lower in the 160 3/4, 2/1w group compared with the preliminary RP2D group (hazard ratio = 0.23, 2-sided *P* = .02), translating to reduced transfusion and dose reduction requirements. The intermittent weekly schedule did not compromise antitumor activity.

**Conclusion:**

The 160 3/4, 2/1w dose was established as an optimized regimen for future camonsertib monotherapy studies offering a substantial reduction in the incidence of anemia without any compromise to efficacy.

**Clinical Trial ID:**

NCT04497116.

Recent U.S. Food and Drug Administration guidance has stressed the need to move away from maximum tolerated dose (MTD)-based approaches to dose selection for targeted oncology therapies. MTD-based approaches (1), originated for chemotherapy agents, are based on toxicities observed during the dose-limiting toxicity (DLT) period (typically comprising the first 3 to 4 weeks of study treatment) and thus, do not take into account later-onset chronic toxicities, particularly relevant to oral targeted agents that can be administered for prolonged durations.

Ataxia telangiectasia and Rad3-related (ATR) kinase inhibitors are a member of the DNA damage response (DDR)-targeting class of small molecules currently in clinical development (2-4). Extensive clinical experience with these and other DDR-targeting agents, such as poly(ADP-ribose) polymerase (PARP) inhibitors, document on-target myelotoxicity as the dominant toxicity (and gastrointestinal toxicities for PARP inhibitors), which can be managed by dose holds and/or reductions and supportive care (5,6).

Camonsertib (RP-3500), a highly potent, selective, oral inhibitor of ATR kinase (7), demonstrated efficacy in biomarker-selected patients with loss-of-function alterations in *ATM* and other DDR genes (8) and displayed a safety profile consistent with other well-characterized DDR inhibitors (9,10). The dominant toxicity, critically important for long-term tolerability, was on-target, mechanism-based anemia in 68% of patients across all dose cohorts (32% grade 3) (8,11).

The first TRESR study trial milestone (Module 1 dose escalation) defined a preliminary monotherapy recommended Phase II dose (RP2D), enabling initiation of the signal-finding Phase IIa portion (dose expansion in prespecified tumor types and genotypes) (8). In parallel, dose optimization continued beyond the preliminary RP2D, including evaluation of an intermittent weekly dosing schedule aimed to mitigate the anemia; avoid unscheduled dose holds, reductions, and transfusions; and enable patients to remain on treatment at pharmacologically active dose levels. Here, we describe a comprehensive approach to optimize the camonsertib monotherapy dose and schedule within the TRESR study. We report on 119 patients, followed for at least 10 months in 3 large dose groups at therapeutic monotherapy doses in the Phase I and Phase IIa parts of the study. Long-term safety, tolerability, and antitumor efficacy profiles were characterized across these groups. Based on a reduced rate of grade 3 anemia with no loss in efficacy, an intermittent weekly schedule (2 weeks on, 1 week off [2/1w]) was selected for future camonsertib monotherapy studies, as an optimized regimen to support prolonged treatment durations.

## Methods

### Study design and patients

The TRESR Phase I and IIa study (NCT04497116) enrolled 154 patients with molecularly selected advanced solid tumors treated with camonsertib monotherapy in dose escalation (Module 1) and expansion (Module 2) cohorts; dose escalation was described previously (8). Three tolerated and efficacious dosing regimens were evaluated with expanded patient numbers to optimize the camonsertib monotherapy dose and schedule. The results described herein include all 119 patients treated at: 1) 120 mg once daily (QD) 3 days on, 4 off (120 3/4; n = 25), 2) 160 3/4 (preliminary RP2D; n = 67 [n = 47 in Module 1; n = 20 in Module 2]), and 3) 160 3/4, 2/1w (n = 27). At the September 13, 2023 data cutoff, all patients had at least 10 months’ follow-up or had discontinued treatment before 10 months. The full inclusion and exclusion criteria for Module 1 were described previously (8). The key eligibility requirements for Modules 1 and 2 are provided in the Supplementary Methods (available online). Baseline demographics (ie, age, sex, race or ethnicity) were collected by the investigator.

The study was conducted in accordance with the Declaration of Helsinki and Council for International Organizations of Medical Sciences International Ethical Guidelines, applicable International Conference on Harmonization, Good Clinical Practice Guidelines, and applicable laws and regulations. All patients provided written informed consent to adhere to the clinical protocol and provided serial blood samples. The protocol was approved by the Institutional Review Board or Ethics Committee at each participating institution.

### Objectives and endpoints

The primary objective of this post hoc analysis was to optimize camonsertib monotherapy dose and schedule based on the evaluation of long-term safety, tolerability, pharmacokinetics, and preliminary antitumor efficacy in the 3 expanded dose groups.

Endpoints for long-term safety and tolerability included treatment-emergent adverse events (TEAEs), serious adverse events (SAEs), and dose interruptions and modifications due to treatment-related adverse events (TRAEs). Efficacy endpoints included overall response rate (Response Evaluation Criteria in Solid Tumors [RECIST] or tumor marker response), clinical benefit rate (RECIST or tumor marker response, or treatment duration of at least 16 weeks without evidence of progression), and progression-free survival (PFS). Molecular responses were characterized by mean variant allele frequency (mVAF) changes in circulating tumor DNA (ctDNA) samples collected longitudinally (an exploratory endpoint).

Camonsertib pharmacokinetics were assessed using a validated liquid chromatography with tandem mass spectrometry analysis of plasma camonsertib concentrations at defined timepoints; pharmacokinetic parameters were derived using noncompartmental analysis and included maximal plasma concentration (C_max_), time to C_max_ (T_max_), area under the curve (AUC), and half-life (t_1/2_). Time above the plasma concentration expected to result in 80% inhibition of tumor checkpoint kinase 1 phosphorylation (pCHK1 IC_80_) based on preclinical xenograft models, a pharmacokinetic driver of efficacy (7), was calculated and compared across the cohorts.

### Statistical analysis

Baseline demographics, disease characteristics, and adverse events (AEs) were summarized with descriptive statistics by dose levels, including all patients treated at these dose levels (safety population). Efficacy endpoints were summarized based on patients with at least 1 post-baseline tumor assessment (efficacy population). Since the two lower doses (120 3/4 and 160 3/4, 2/1w) had comparable average dose intensity in each cycle, the main comparison was pairwise comparison between each of these lower dose levels versus the highest dose intensity 160 3/4 dose group. The estimated cumulative incidence rate over time was provided for the grade 3+ anemia, dose reductions due to AEs, or grade 3+ neutropenia and thrombocytopenia. The estimated cumulative incidence rate was based on the Fine and Gray method, counting early discontinuation as a competing event. Those patients with ongoing treatment were censored at the data cutoff. The relative risk of the event was estimated by the hazard ratio (HR) between each of the two lower dose levels versus the highest dose (160 3/4), under the framework of Cox proportional hazard models. Additional baseline predictors for the onset for grade 3 treatment-related anemia were explored with multivariate Cox regression models. Nominal *P*-values (2-sided) were provided in all cases for hypothesis-generating purposes, with a significance level of .05, without adjusting for multiplicity.

### ctDNA analysis

Blood was collected pretreatment and on day 1 of each cycle. Cell-free DNA was isolated and sequenced, using a commercially available targeted panel (8). Germline and variants derived from clonal hematopoiesis were filtered by comparison with targeted matched peripheral blood mononuclear cells sequencing. Artifacts and suspected germline variants were removed by manual curation. To monitor the molecular changes in ctDNA with camonsertib treatment, the mVAF was calculated for each timepoint; molecular response was defined as a best response of greater than or equal to a 50% reduction in mVAF from baseline (8).

## Results

### Camonsertib dose and schedule optimization

The TRESR study initially evaluated 2 different intermittent dosing schedules. The 3/4 schedule was selected over a 5-days-on, 2-days-off (5/2) schedule for further evaluation (8). Supplementary Figure 1 (available online) shows a schematic of the dose finding and optimization strategy.

This article focuses on 119 patients treated with camonsertib monotherapy within 3 large dose cohorts on the 3/4 schedule: 160 mg QD (the initial preliminary RP2D; n = 67), and two step-down dose levels of 120 mg QD (n = 25), and 160 mg QD on a 2/1w schedule (n = 27). The expansion at the preliminary RP2D of 160 3/4 afforded a large cohort to further characterize long-term tolerability. Doses of more than 100 mg daily were projected as biologically active by pharmacokinetic and pharmacodynamic data, and clinical activity was confirmed at these doses (8). Baseline patient characteristics for the 3 dose groups are shown in Table 1. The most common tumor types were ovarian (n = 23), breast (n = 15), pancreatic (n = 14), and prostate (n = 14); the most frequent enrollment genes were *ATM* (n = 38), *BRCA1* (n = 28), *BRCA2* (n = 15), *SETD2* (n = 10), and *CDK12* (n = 7).

**Table 1.** Baseline characteristics of patients in each dose group

	120 3/4	160 3/4	160 3/4, 2/1w	All patients
	(n = 25)	(n = 67)	(n = 27)	(N = 119)
Sex, no. (%)				
Male	10 (40.0)	25 (37.3)	11 (40.7)	46 (38.7)
Female	15 (60.0)	42 (52.7)	16 (59.3)	73 (61.3)
Age, years				
Median (range)	64 (39–76)	61 (36–77)	68 (30–77)	63 (30–77)
65 and older, no. (%)	12 (48.0)	23 (34.3)	16 (59.3)	51 (42.9)
ECOG status, no. (%)				
0	11 (44.0)	34 (50.7)	17 (63.0)	62 (52.1)
1	14 (56.0)	33 (49.3)	10 (37.0)	57 (47.9)
Lines of prior systemic therapy, no. (%)				
3 or less	12 (48.0)	45 (67.2)	20 (74.1)	77 (64.7)
4 or more	13 (52.0)	22 (32.8)	7 (25.9)	42 (35.3)
Prior platinum, no. (%)	14 (56.0)	43 (64.2)	22 (81.5)	79 (66.4)
Prior PARP inhibitor, no. (%)	8 (32.0)	24 (35.8)	10 (37.0)	42 (35.3)
Tumor type, no. (%)				
Ovarian	5 (20.0)	11 (16.4)	7 (25.9)	23 (19.3)
Breast	2 (8.0)	9 (13.4)	4 (14.8)	15 (12.6)
Pancreas	0	10 (14.9)	4 (14.8)	14 (11.8)
Prostate	6 (24.0)	6 (9.0)	2 (7.4)	14 (11.8)
Other^a^	12 (48.0)	31 (46.3)	10 (37.0)	53 (44.5)
Enrollment gene, no. (%)				
*ATM*	5 (20.0)	25 (37.3)	8 (29.6)	38 (31.9)
*BRCA1*	4 (16.0)	15 (22.4)	9 (33.3)	28 (23.5)
*BRCA2*	4 (16.0)	8 (11.9)	3 (11.1)	15 (12.6)
*SETD2*	3 (12.0)	6 (9.0)	1 (3.7)	10 (8.4)
*CDK12*	3 (12.0)	3 (4.5)	1 (3.7)	7 (5.9)
*NBN*	0	3 (4.5)	2 (7.4)	5 (4.2)
*PALB2*	3 (12.0)	2 (3.0)	0	5 (4.2)
Other^b^	3 (12.0)	5 (7.5)	3 (11.1)	11 (9.2)
Baseline hematology, median				
Hemoglobin, g/dL	11.3	11.9	11.9	11.9
Neutrophils, K/μL	3.9	4.2	3.7	4.1
Platelets, K/μL	215	257	231	231

a Other includes colorectal (n = 8), soft-tissue sarcoma (n = 8), non-small cell lung cancer (n = 7), kidney (n = 4), bile duct (n = 4), endometrial (n = 3), head and neck (n = 3), gastrointestinal (n = 3), and other less frequent tumor types (n = 13). 2/1w = 2 weeks on, 1 week off; 120 3/4 = 120 mg QD 3 days on, 4 days off; 160 3/4 = 160 mg QD 3 days on, 4 days off; ECOG = Eastern Cooperative Oncology Group; K/μL = thousand cells per microliter; PARP = poly (ADP-ribose) polymerase; QD = once daily.

b Other includes *CHEK2* (n = 2), *RAD51B* (n = 2), *RAD51C* (n = 4), and *RNASEH2* (n = 3).

Pharmacokinetic parameters of camonsertib across the 3 dose groups are included in Supplementary Figure 2 (available online); the dose normalized C_max_, and area under the concentration-time curve from dosing to time t (AUC_0-t_) were similar across the groups (on both cycle 1, day 1 and 3; Supplementary Figure 2, Supplementary Table 1, available online). Comparable increases in pharmacodynamic markers (γ-H2AX and p-KAP1; associated with inhibition of the ATR checkpoint) were observed in paired biopsies taken before and during treatment at cycle 2, day 10 across the 3 dose groups (7,8).

### Safety and tolerability

**Figure 1.**
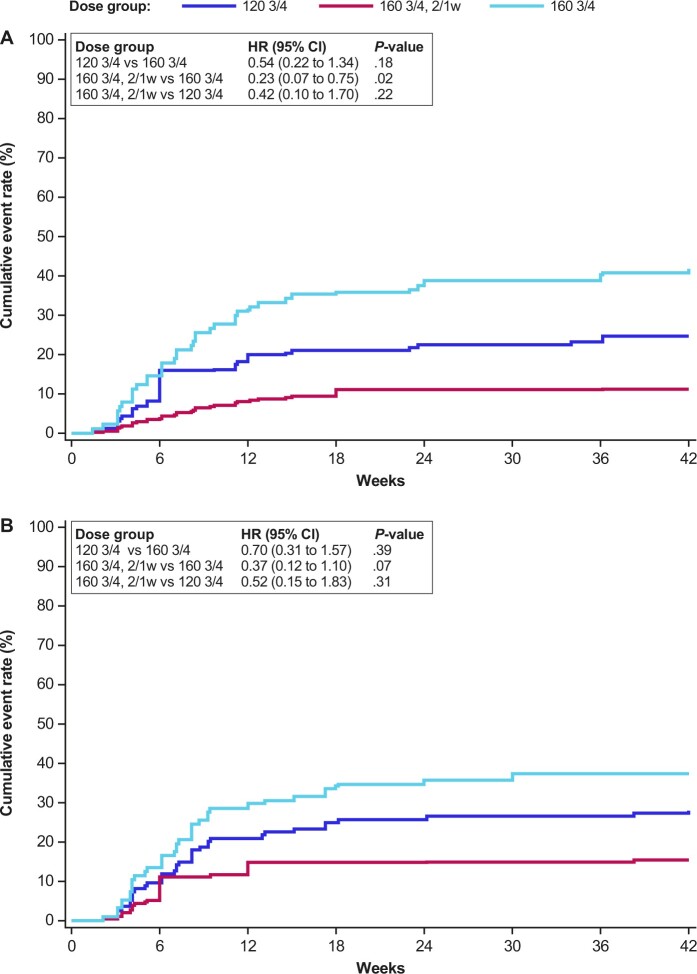
Cumulative incidence of grade 3 anemia and dose reductions over time. **A)** Treatment-related grade 3 anemia according to dose and schedule. **B)** Dose reductions due to treatment-emergent adverse events according to dose and schedule. HR and *P*-values presented here were not adjusted for other factors. 2/1w = 2 weeks on, 1 week off; 120 3/4 =120 mg QD 3 days on, 4 days off; 160 3/4 = 160 mg QD 3 days on, 4 days off; CI = confidence interval; HR = hazard ratio; QD = once daily.

The median time on treatment was 11.4 weeks (range 0.4–117.1 weeks), similar across the 3 dose groups. Anemia (all grades) was the most common on-target TRAE across the 3 dose groups (120 3/4: 72.0% [18/25]; 160 3/4: 70.1% [47/67]; 160 3/4, 2/1w: 55.6% [15/27]). The incidence of grade 3 anemia was lower in patients treated at 160 3/4, 2/1w (11.1% [3/27]) compared with both continuous weekly schedules (120 3/4: 24.0% [6/25]; 160 3/4: 41.8% [28/67]; Table 2). Figure 1, A depicts the cumulative incidence of grade 3 anemia development over time for the 3 dose groups. The risk of developing grade 3 anemia for patients treated at 160 3/4, 2/1w was significantly reduced compared with that for patients at the same daily dose administered on the continuous weekly schedule (HR 0.23, *P* = .02), whereas the decreased weekly dose of 120 3/4 had a moderately reduced risk of grade 3 anemia vs 160 3/4 (HR 0.54, *P* = .18). Prolonged treatment duration resulted in a continued increase in the incidence of grade 3-related anemia within the continuous weekly dosing groups (new incidences as late as 36 weeks), whereas no additional patients in the 160 3/4, 2/1w group developed grade 3 anemia after 18 weeks of therapy (Figure 1, A). Evaluation of hematologic parameters demonstrated that the week off treatment for patients in the 2/1w dosing group provided sufficient time for a full rebound in monocyte and reticulocyte counts before the start of the next dosing cycle, consistent with the lower rate of clinically significant anemia in that group (Supplementary Figure 3, available online) (12,13).

**Table 2.** TRAE in at least 5% of patients by dose group

	120 3/4		160 3/4		160 3/4, 2/1w		All patients	
	(n = 25)		(n = 67)		(n = 27)		(N = 119)	
Preferred term	All grade	Grade 3+	All grade	Grade 3+	All grade	Grade 3+	All grade	Grade 3+
	no. (%)	no. (%)	no. (%)	no. (%)	no. (%)	no. (%)	no. (%)	no. (%)
Any event	23 (92.0)	8 (32.0)	58 (86.6)	32 (47.8)	24 (88.9)	7 (25.9)	105 (88.2)	47 (39.5)
Anemia	18 (72.0)	6 (24.0)	47 (70.1)	28 (41.8)	15 (55.6)	3 (11.1)	80 (67.2)	37 (31.1)
Fatigue	8 (32.0)	1 (4.0)	21 (31.3)	2 (3.0)	7 (25.9)	0	36 (30.3)	3 (2.5)
Neutropenia	6 (24.0)	2 (8.0)	23 (34.3)	12 (17.9)	6 (22.2)	4 (14.8)	35 (29.4)	18 (15.2)^a^
Nausea or vomiting	9 (36.0)	0	17 (25.4)	0	5 (18.5)	0	31 (26.1)	0
Thrombocytopenia	4 (16.0)	1 (4.0)	13 (19.4)	6 (9.0)	8 (29.6)	0	25 (21.0)	7 (5.8) ^a^
Decreased appetite	9 (36.0)	0	8 (11.9)	0	1 (3.7)	0	18 (15.1)	0
Diarrhea	6 (24.0)	0	8 (11.9)	0	5 (18.5)	0	19 (16.0)	0
Leukopenia	4 (16.0)	0	7 (10.4)	4 (6.0)	3 (11.1)	1 (3.7)	14 (11.8)	5 (4.2)
Dyspnea	1 (4.0)	0	7 (10.4)	0	0	0	8 (6.7)	0
Dysgeusia	2 (8.0)	0	4 (6.0)	0	0	0	6 (5.0)	0
Constipation	2 (8.0)	0	3 (4.5)	0	1 (3.7)	0	6 (5.0)	0
Rash maculo-papular	1 (4.0)	0	4 (6.0)	0	1 (3.7)	0	6 (5.0)	0
ALT or AST increased	0	0	3 (4.5)	0	2 (7.4)	0	5 (4.2)	0
Blood ALP increased	0	0	2 (3.0)	0	2 (7.4)	0	4 (3.4)	0
Weight decreased	1 (4.0)	0	0	0	2 (7.4)	0	3 (2.5)	0

a There were 5 incidences of grade 4 TRAE: 160 3/4 group: grade 4 thrombocytopenia (n = 1), grade 4 neutropenia (n = 3); 160 3/4, 2/1w group: grade 4 neutropenia (n = 1). 2/1w = 2 weeks on, 1 week off; 120 3/4 = 120 mg QD 3 days on, 4 days off; 160 3/4 = 160 mg QD 3 days on, 4 days off; ALT = alanine aminotransferase; AST = aspartate aminotransferase; ALP = alkaline phosphatase; QD = once daily; TRAE = treatment-related adverse events.

Given the heterogeneity in baseline characteristics across the 3 dose groups, a multivariate Cox regression model was used to assess baseline predictors for grade 3 anemia (Supplementary Table 2, available online). Besides baseline hemoglobin level, patients who were more heavily pretreated (more than 3 prior regimens) had an increased risk of grade 3 anemia (HR 2.32, *P* = .02) compared with those less heavily pretreated. Patients with an Eastern Cooperative Oncology Group (ECOG) Performance Status score of 1 (vs 0) also had a slightly higher risk of grade 3 anemia (HR 1.85, *P* = .07). After adjusting for baseline hemoglobin, ECOG score, and number of prior regimens (more than 3 vs 3 or less), both the 120 3/4 (HR 0.35, *P* = .02) and 160 3/4, 2/1w (HR 0.31, *P* = .02) groups showed a statistically significant decrease in anemia rates compared with the 160 3/4 group.

As a result of the reduced rate of grade 3 anemia in patients in the 160 3/4, 2/1w group, fewer treatment interventions were required, including fewer red blood cell transfusions due to grade 3-related anemia (7.4% [2/27] in the 160 3/4, 2/1w group vs 20.0% [5/25] and 28.4% [19/67] in the 120 3/4 and 160 3/4 groups, respectively; Table 3). Furthermore, the incidence of AE-related dose reductions was numerically lower for patients on the 160 3/4, 2/1w schedule (14.8% [4/27]), compared with the continuous weekly schedules (120 3/4: 28.0% [7/25]; 160 3/4: 37.3% [25/67]; Table 3 and Figure 1, B).

**Table 3.** Dose reductions and transfusions by dose group

	120 3/4	160 3/4	160 3/4, 2/1w	All patients
	(n = 25)	(n = 67)	(n = 27)^a^	(N = 119)
Duration of treatment, weeks				
Mean (SD)	24.2 (26.7)	15.3 (13.5)	27.3 (34.3)	19.9 (23.1)
Median	11.4	11.4	11.1	11.4
Range	2.4–94.3	0.4–64.6	0.4–117.1	0.4–117.1
Dose reduction due to AE, no. (%)	7 (28.0)	25 (37.3)	4 (14.8)	36 (30.3)
Subjects with RBC transfusion (for grade 3 anemia), no. (%)	5 (20.0)	19 (28.4)	2 (7.4)	26 (21.8)

a One patient from the 160 3/4, 2/1w group had dose reduced to 120 3/4, 2/1w due to a medical history of grade 2 anemia (not a TRAE). 2/1w = 2 weeks on, 1 week off; 120 3/4 = 120 mg QD 3 days on, 4 days off; 160 3/4 = 160 mg QD 3 days on, 4 days off; AE = adverse event; QD = once daily; RBC = red blood cell; SD = standard deviation; TRAE, treatment-related AE.

In contrast to the lower incidence of grade 3 anemia on the 2/1w schedule, the incidence of grade 3+ neutropenia was similar in patients treated at 160 3/4 on either the continuous weekly (12/67; 17.9%) or 2/1w (4/27; 14.8%) schedules; patients treated at 120 3/4 had a numerically lower incidence (2/25; 8.0%; Table 2). The onset of grade 3+ neutropenia or thrombocytopenia tended to occur earlier during treatment (in most cases, within the first 6 weeks) compared to grade 3 anemia, which typically occurred at later cycles (ie, after 6 weeks; Supplementary Figure 4, available online).

The most common nonhematologic TRAE across all dose groups was fatigue (all grades: 30.3%), which was mostly low grade (grade 3: 2.5%), and similar across groups (Table 2). Gastrointestinal toxicities were all low grade (no grade 3+) with comparable frequency (nausea or vomiting: 26.1%; diarrhea: 16.0%) across the dose groups.

### Antitumor activity

**Figure 2.**
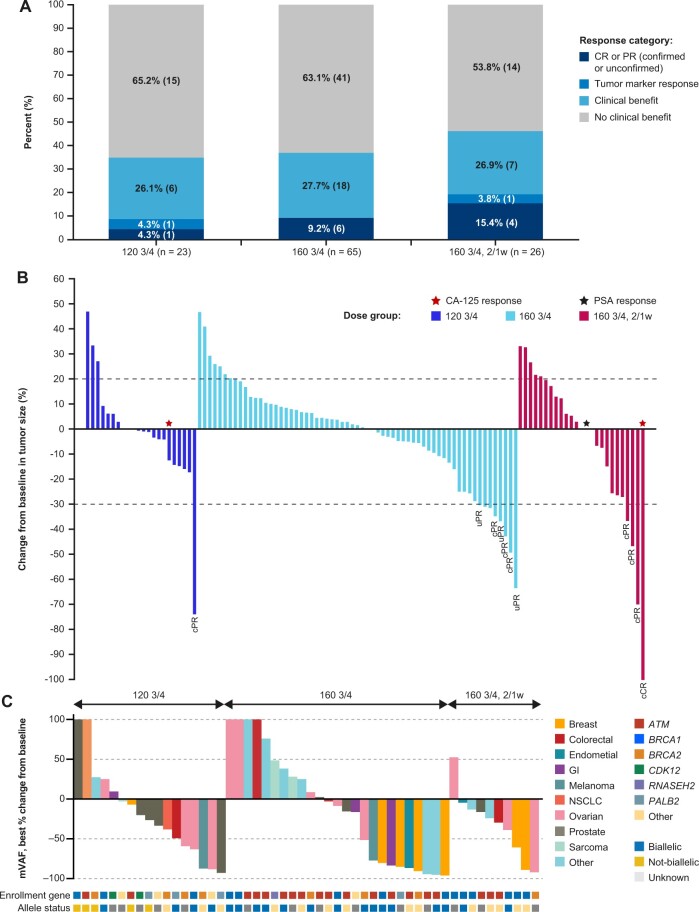
Efficacy by dose group. **A)** Overall response. **B)** Change from baseline in tumor size by dose group. **C)** ctDNA responses by dose group. 2/1w = 2 weeks on, 1 week off; 120 3/4 =120 mg QD 3 days on, 4 days off; 160 3/4 = 160 mg QD 3 days on, 4 days off; CA-125 = cancer antigen 125; cCR = confirmed CR; cPR = confirmed PR; CR = complete response; ctDNA = circulating tumor DNA; GI = gastrointestinal; mVAF = mean variant allele frequency; NSCLC = non-small cell lung cancer; PR = partial response; PSA = prostate-specific antigen; QD = once daily; uPR = unconfirmed PR.

**Figure 3.**
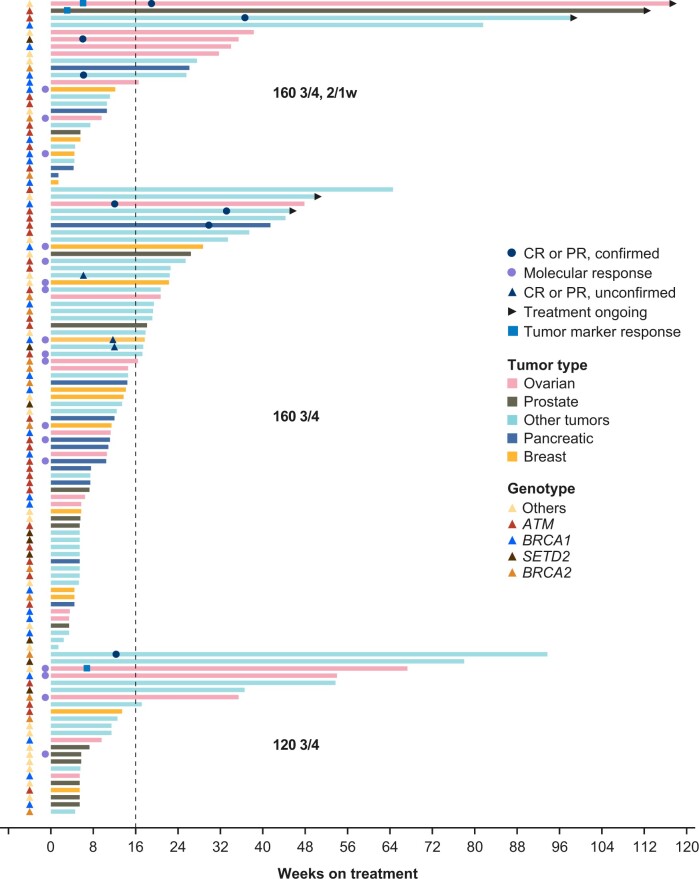
Swim plot of duration of treatment and responses to camonsertib by dose group. 2/1w = 2 weeks on, 1 week off; 120 3/4 =120 mg QD 3 days on, 4 days off; 160 3/4 = 160 mg QD 3 days on, 4 days off; CR = complete response; PR = partial response; QD = once daily.

Clinical activity, determined by RECIST v.1.1 and/or tumor marker (eg, prostate-specific antigen, cancer antigen-125 [CA-125]) responses was observed in all 3 dose groups with overall response rates of 8.7% (120 3/4), 9.2% (160 3/4), and 19.2% (160 3/4, 2/1w; Figures 2, A and B; Table 4; Supplementary Table 3, available online). Clinical benefit rates were 34.8% (120 3/4), 36.9% (160 3/4), and 46.2% (160 3/4, 2/1w). Prolonged duration of treatment with RECIST v.1.1 stable disease was observed in all 3 groups (Figure 3). In the 160 3/4, 2/1w group, 42% (11/26) of efficacy-evaluable patients remained on treatment for more than 24 weeks vs 32% and 16% in the 120 3/4 and 160 3/4 dose groups, respectively. Patients requiring dose reductions to 120 3/4, 2/1w (n = 20; Supplementary Figure 5, available online) also had prolonged treatment at the reduced dose (Supplementary Figure 6, available online). Median PFS was similar across the 3 groups (overlapping 95% confidence intervals): 13.4 (120 3/4), 16.1 (160 3/4), and 14.7 weeks (160 3/4, 2/1w); at 24 weeks, the estimated progression-free rate was the lowest for the 160 3/4 regimen, favoring the lower dose groups (Table 4). Due to the small sample size, the difference in PFS was not statistically significant. In the subset of patients monitorable by ctDNA (n = 52), mVAF reductions were observed across all 3 dose groups with molecular response rates of 24% (120 3/4), 40% (160 3/4), and 30% (160 3/4, 2/1w; Figure 2, C).

**Table 4.** Efficacy summary across the 3 dose groups

Endpoint or category	120 3/4	160 3/4	160 3/4, 2/1w	All patients
	(n = 23)	(n = 65)	(n = 26)	(n = 114)
Best response by RECIST v1.1, no. (%)				
cCR	0	0	1 (3.8)	1 (0.9)
cPR	1 (4.3)	3 (4.6)	3 (11.5)	7 (6.1)
uCR or uPR	0	3 (4.6)	0	3 (2.6)
Overall response (RECIST only), no. (%, 95% CI)	1 (4.3, 0.1 to 21.9)	6 (9.2, 3.5 to 19.0)	4 (15.4, 4.4 to 34.9)	11 (9.6, 4.9 to 16.6)
Overall response (RECIST or tumor marker), no. (%, 95% CI)	2 (8.7, 1.1 to 28.0)	6 (9.2, 3.5 to 19.0)	5 (19.2, 6.6 to 39.4)	13 (11.4, 6.2 to 18.7)
Clinical benefit,^a^ no. (%, 95% CI)	8 (34.8, 16.4 to 57.3)	24 (36.9, 25.3 to 49.8)	12 (46.2, 26.6 to 66.6)	44 (38.6, 29.6 to 48.2)
Median^b^ PFS, weeks (95% CI)	13.4 (7.00 to 37.00)	16.1 (11.43 to 17.71)	14.7 (6.14 to 27.29)	14.7 (12.14 to 17.71)
PFS at 24 weeks, % (95% CI)	38.9 (18.0 to 59.5)	25.2 (14.5 to 37.4)	45.0 (25.3 to 62.8)	33.1 (24.0 to 42.5)
Molecular response (ctDNA reduction of 50% or more)				
Evaluable ctDNA for monitoring	17	25	10	52
Molecular response, response/evaluable (%)	4/17 (23.5)	10/25 (40.0)	3/10 (30.0)	17/52 (32.7)

a Defined as overall response, or duration of treatment of at least 16 weeks without progressive disease. 2/1w = 2 weeks on, 1 week off; 120 3/4 = 120 mg QD 3 days on, 4 days off; 160 3/4 = 160 mg QD 3 days on, 4 days off; CA-125 = cancer antigen 125; cCR = confirmed complete response; CI = confidence interval; cPR = confirmed partial response; ctDNA = circulating tumor DNA; PFS = progression-free survival; PSA = prostate-specific antigen; QD = once daily; RECIST = Response Evaluation Criteria in Solid Tumors; uCR, unconfirmed complete response; uPR = unconfirmed partial response.

Across the 114 efficacy evaluable patients in the 3 camonsertib monotherapy dose groups, responses were observed in patients enrolled with alterations in *ATM* (n = 4), *BRCA1* (n = 3), *RAD51C* (n = 3), *SETD2* (n = 2), and *BRCA2* (n = 1; Table 5; Supplementary Table 3, available online). Consistent with prior results (8), RECIST responses in patients with tumors harboring ATM alterations occurred later (median: 33.1 weeks) compared to patients with *BRCA*-altered tumors (median: 11.9 weeks); this updated dataset includes 2 patients with g*ATM* (1 pancreatic, 1 stomach) enrolled in the Phase IIa portion of the study with initial responses at 30 and 33 weeks, respectively (Figure 3, Table 5, Supplementary Table 3, available online). Responses in tumors harboring genomic alterations in *RAD51C* and *SETD2* were notable given the underrepresentation of these genotypes in the study. Three out of 4 patients with *RAD51C*-altered tumors had a RECIST 1.1 (1 complete response, 1 unconfirmed partial response) or CA-125 (n = 1) response; 4/4 had clinical benefit (treatment durations: 23, 38, 67, and 117+ weeks). Two out of 9 patients with *SETD2*-altered tumors had a RECIST 1.1 response, and 4/9 had clinical benefit (3 with treatment durations of more than 35 weeks [range 35–78]).

**Table 5.** Response summary by genotype (combined across the 3 dose groups, N = 114)

Endpoint or category	*ATM*	*BRCA1/2*	*CDK12*	*NBN*	*PALB2*	*RAD51B/C^a^*	*SETD2*	Others^b^
	(n = 36)	(n = 42)	(n = 6)	(n = 5)	(n = 5)	(n = 6)	(n = 9)	(n = 5)
Best response by RECIST v1.1, no. (%)								
cCR	0	0	0	0	0	1 (16.7)	0	0
cPR	3 (8.3)	3 (7.1)	0	0	0	0	1 (11.1)	0
uCR or uPR	0	1 (2.4)	0	0	0	1 (16.7)	1 (11.2)	0
Overall response (RECIST only), no. (%)	3 (8.3)	4 (9.5)	0	0	0	2 (33.3)	2 (22.2)	0
Overall response (RECIST or tumor marker), no. (%)	4 (11.1)	4 (9.5)	0	0	0	3 (50.0)^c^	2 (22.2)	0
Clinical benefit rate,^d^ no. (%)	15 (41.7)	14 (33.3)	0	2 (40.0)	1 (20.0)	6 (100)	4 (44.4)	2 (40.0)
Median^e^ PFS, weeks	17.7	12.6	11.6	12.1	7.0	45.5	18.0	7.0
Median^e^ duration of treatment, weeks (min, max)	11.1 (4.3, 112.3)	11.8 (1.4, 93.7)	6.4 (3.4, 13.7)	12.4 (1.4, 31.7)	5.7 (5.4, 22.3)	52.8 (17.9, 117.1)	13.4 (2.4, 78.0)	5.7 (5.3, 33.4)
Median time to response, weeks (min, max)	33.1 (29.9, 36.6)	11.9 (6.1, 12.3)	–	–	–	12.6 (6.1, 19.0)	9.0 (6.0, 12.0)	–
Median duration of response,^f^ weeks (min, max)	24.3 (12.3, 36.3)	36.0 (20.3, 74.4)	–	–	–	36.0 (36.0, 36.0)	23.1 (23.1, 23.1)	–
Molecular response (ctDNA reduction of 50% or more)								
Evaluable ctDNA for monitoring	19	22	2	1	4	1	0	3
Molecular response, n/m (%)	5/19 (26.3)	9/22 (40.9)	0/2 (0)	0/1 (0)	2/4 (50.0)	1/1 (100)	N/A	0/3 (0)

a *RAD51B* (n = 2*), RAD51C* (n = 4). cCR = confirmed complete response; CI = confidence interval; cPR = confirmed partial response; ctDNA = circulating tumor DNA; m = no. evaluable ctDNA for monitoring; max = maximum; min = minimum; N/A = not applicable; PFS = progression-free survival; RECIST = Response Evaluation Criteria in Solid Tumors; uCR = unconfirmed complete response; uPR = unconfirmed partial response.

b Other genotypes include *CHEK2* (n = 2) and *RNASEH2* (n = 3).

c All 3 patients with responses had *RAD51C* alterations.

d Defined as overall response or duration of treatment of at least 16 weeks without progressive disease.

e Kaplan–Meier method.

f Applicable to patients with a cCR or cPR.

## Discussion

We present a novel and comprehensive dose-finding strategy for the oral ATR inhibitor camonsertib, from the Phase I and IIa TRESR trial. After choosing a preliminary RP2D (160 3/4 on a continuous weekly schedule) based on the observation of DLTs during the dose-escalation phase, a comprehensive dose-optimization analysis was conducted (Supplementary Table 4 and Supplementary Figure 7, available online, highlight the differences between this analysis and the previously reported analyses in Yap et al.) (8). Further enrollment at the preliminary RP2D proceeded with concurrent analysis of long-term tolerability and efficacy against 2 alternative dose options of lower dose intensity: a reduced daily dose or introduction of 1 week off treatment each cycle.

Safety, tolerability, and antitumor activity data were comprehensively assessed in 119 patients (114 efficacy evaluable) in these 3 dose groups after a follow-up time of at least 10 months. A camonsertib monotherapy dose of 160 mg QD 3/4 administered on a 2/1w schedule demonstrated substantial tolerability improvement, reduction of related grade 3 anemia, blood transfusions, and dose modifications, compared with the 160 mg QD 3/4 continuous weekly schedule. The modified schedule did not impact the rates of the lower-grade nonhematologic toxicities (eg, fatigue, gastrointestinal toxicity).

Although the 2 alternative dose groups were not directly compared, the 120 3/4 group had similar requirements for dose reductions as the preliminary R2PD of 160 3/4. The rebound in monocytes and reticulocytes, indicative of bone marrow recovery, during the week off treatment may explain the lower rates of anemia for the 2/1w schedule.

Our strategy to critically evaluate both the dosage and schedule, and to assure the final decision based on long-term follow-up, aligns with the US Food and Drug Administration guidelines (Project Optimus), which encourages robust dose-finding in early clinical development to optimize safety and tolerability (1).

The Methodology for the Development of Innovative Cancer Therapies Taskforce guidelines recommend shifting from an RP2D definition that is frequently close to the MTD to a recommended dose range that considers the target population, drug mechanisms of action, and longitudinal toxicity endpoints (14).

For molecular targeted therapies, a “more is better” paradigm may not apply, with several examples of agents in clinical practice used at lower doses than those established in registration trials requiring dose optimization in retrospective evaluation or post-registration studies (15).

Here, we report comparable antitumor activity but different toxicity profiles in the 3 active dose groups of camonsertib monotherapy. We propose the intermittent weekly dose schedule as it results in better camonsertib tolerability with no decrease in antitumor activity in the population studied. To avoid dose reductions of camonsertib to levels with unexplored biological activity, we provide comprehensive safety and activity data for the 3 active groups to tailor both dose and schedule for patients. Based on these data, treatment personalization within the active dose range could include 120 3/4 for patients with a predominant toxicity of neutropenia; escalation to 160 3/4 could be an approach for patients who tolerate camonsertib without clinically significant anemia and may benefit from a higher-intensity dose.

To further enable a personalized approach, we developed a nomogram based on early hematological changes to predict the degree of hemoglobin decline by week 4 (13). This tool may aid clinicians with dose and schedule adjustments to avoid blood transfusions and unscheduled dose interruptions (13).

Limitations of this study include the heterogenous patient population and lack of randomization, which are typical characteristics of Phase I trials. Patients treated within the 3 dose groups had heterogeneous baseline characteristics and clinical history, including previous lines of treatment and exposure to PARP inhibitors or platinum chemotherapy agents. Accounting for baseline heterogeneity, Cox regression models showed an independent association of the alternative camonsertib doses with a lower risk of grade 3 anemia, supporting the implementation of the alternative dosing schedule.

Given the heterogeneity of tumor types, genotypes, and other features such as allelic status, conclusions regarding superiority in efficacy for any of the 3 dose groups cannot be made. Herein, we extend the results previously reported (8) by evaluating antitumor activity in an expanded patient population treated at the preliminary RP2D and 2 efficacious step-down doses. Additional late responses (at 30 and 33 weeks) were reported in patients with g*ATM*. Although the patient numbers were small, the clinical benefit for patients with less common genomic alterations, namely *RAD51C* and *SETD2*, became apparent. As reported in Yap et al. (8), tumors with biallelic loss remained associated with longer PFS and duration of treatment. The robustness of the efficacy signal at the selected dose of 160 3/4, 2/1w is being confirmed in later phase trials and more homogeneous patient populations (NCT04589845, NCT03337698).

In conclusion, based on the lower risk of grade 3 anemia (the dominant camonsertib toxicity) and the preservation of antitumor activity, the camonsertib monotherapy dose of 160 3/4 administered on a 2/1w schedule was selected as the optimized regimen for future pivotal studies.

## Supplementary Material

djae098_Supplementary_Data

## Data Availability

To minimize the risk of patient reidentification, data will only be shared upon reasonable request. For eligible studies, qualified researchers may request access to individual patient-level clinical data through a data request platform. At the time of writing, this request platform is Vivli (https://vivli.org/ourmember/roche/). Datasets can be requested 18 months after a clinical study report has been completed and, as appropriate, once the regulatory review of the indication or drug has been completed. Access to patient-level data from this trial can be requested and will be assessed by an independent review panel, which decides whether the data will be provided. Once approved, the data are available for up to 24 months. For up-to-date details on Roche’s Global Policy on the Sharing of Clinical Information and how to request access to related clinical study documents, see https://go.roche.com/data_sharing. Anonymized records for individual patients across more than one data source external to Roche cannot, and should not, be linked owing to a potential increase in risk of patient reidentification.
