# Supplementary material for: Ataxia telangiectasia and Rad3-related (ATR) inhibitor camonsertib dose optimization in patients with biomarker-selected advanced solid tumors (TRESR study)
Source: J Natl Cancer Inst. 2024 May 6;116(9):1439–49. doi: 10.1093/jnci/djae098 (PMC11378309; doi:10.1093/jnci/djae098)
Supplement: djae098_Supplementary_Data [file djae098_supplementary_data.pdf]

Supplementary Material

Supplementary Methods

Patient eligibility

Key inclusion criteria included age of 18 years or older at the time of consent, Eastern Cooperative Oncology Group (ECOG) Performance Status score 0 or 1, baseline hemoglobin of 9.5 g/dL or more (10 g/dL for Module 2), histologically confirmed solid tumors resistant or refractory to standard treatment, and/or patients intolerant to standard therapy. No red blood cell or platelet transfusions, or growth factors were allowed within 7 days of the first dose of study drug (14 days for Module 2). For enrollment in Module 1 deleterious/likely deleterious alterations in at least one of the following genes was required: *ATM*, *ATRIP*, *BRCA1*, *BRCA2*, *CDK12*, *CHTF8*, *FZR1*, *MRE11*, *NBN*, *PALB2*, *RAD17*, RAD50, *RAD51B/C/D*, *REV3L*, *RNASEH2A*, *RNASEH2B*, *SETD2*, or other genes agreed with the sponsor and investigator. Patients enrolled in Module 2 were enrolled into 1 of 3 arms: arm 1) Selected tumor types (estrogen receptor-positive/ human epidermal growth factor receptor 2-negative breast, ampullary, pancreas, prostate, bile duct, and gastroesophageal junction cancers) with likely pathogenic or pathogenic germline *ATM* mutations (n = 5); arm 2) Leiomyosarcoma tumors with RNASEH2 loss or deleterious or likely deleterious *BRCA2* mutations (n = 1); or arm 3) Tumors with other ataxia telangiectasia and Rad3-related (ATR) inhibitor-sensitizing biomarkers: *ATRIP, CHTF8, FZR1, MRE11, NBN, RAD17, RAD50, RAD51B/C/D, REV3L, SETD2*, and other genes as agreed between the sponsor and investigator (n = 14). Genetic eligibility was centrally confirmed and annotated by the University of Texas MD Anderson Cancer Center Precision Oncology Decision Support group (1). For Module 2 arms 1 and 2 (n = 6), patients may have had up to 2 prior nonhormonal

anticancer regimens in the metastatic setting (no limitation on number of prior lines for Module 1 or Module 2, arm 3). Key exclusion criteria for both modules included treatment with chemotherapy, small molecule, or biologic anticancer therapy within 14 days prior to first dose of the study drug, or prior therapy with an ATR or DNA-dependent protein kinase inhibitor.

References

1. Johnson A, Zeng J, Bailey AM, et al. The right drugs at the right time for the right patient: the MD Anderson precision oncology decision support platform. *Drug Discov Today.* 2015;20(12):1433-8.

Supplementary Table 1. Pharmacokinetic parameters for the 3 dose groups

Statistical Parameter	Module 1, cycle 1, day 1							Module 1, cycle 1, day 3						
	$t_{1/2}$ (hr)	C_{max} (μg/mL)	C_{max}/dose ([μg/mL]/mg)	AUC_{last} (μg.hr/mL)	AUC_{last}/dose ([μg.hr/mL]/mg)	AUC_{INF} (μg.hr/mL)	Time above IC_{80} (hr)	$t_{1/2}$ (hr)	C_{max} (μg/mL)	C_{max}/dose ([μg/mL]/mg)	AUC_{last} (μg.hr/mL)	AUC_{last}/dose ([μg.hr/mL]/mg)	AUC_{INF} (μg.hr/mL)	Time above IC_{80} (hr)
**120 3/4**														
No.	24	25	25	25	25	24	25	18	19	19	19	19	18	19
Mean	6.33						7.11	5.90						7.47
SD	2.08						3.91	1.71						4.90
CV%	32.8						55.0	29.0						65.6
Geo. mean		5.36	0.0446	28.4	0.237	32.0			4.98	0.0415	28.5	0.237	29.4	
Geo. SD		1.58	1.58	1.74	1.74	1.74			1.42	1.42	1.71	1.71	1.76	
Geo. CV%		48.1	48.1	59.6	59.6	59.7			36.5	36.5	57.4	57.4	61.6	
**160 3/4**														
No.	46	47	47	47	47	46	45	41	44	44	44	44	41	39
Mean	5.74						9.43	6.00						10.3
SD	1.73						4.52	1.52						5.35
CV%	30.2						47.9	25.3						52.0
Geo. mean		7.40	0.0462	38.8	0.243	42.6			7.88	0.0493	42.8	0.268	47.5	

Geo. SD		1.48	1.48	1.64	1.64	1.67			1.40	1.40	1.65	1.65	1.72	
Geo. CV%		40.7	40.7	53.0	53.0	54.8			34.9	34.9	53.7	53.7	58.2	
**160 3/4, 2/1w**														
No.	26	27	27	27	27	26	23	24	25	25	25	25	24	24
Mean	5.55						9.55	6.18						11.3
SD	1.36						4.28	1.92						6.15
CV%	24.6						44.8	31.1						54.2
Geo. mean		7.43	0.0464	41.0	0.256	47.4			7.58	0.0474	46.8	0.292	52.0	
Geo. SD		1.43	1.43	1.47	1.47	1.59			1.55	1.55	1.70	1.70	1.77	
Geo. CV%		36.8	36.8	39.9	39.9	49.3			46.0	46.0	56.8	56.8	62.4	

2/1w = 2 weeks on, 1 week off; 120 3/4 =120 mg QD 3 days on, 4 days off; 160 3/4 = 160 mg QD 3 days on, 4 days off; AUC_{INF} = area under the plasma concentration-time curve from time 0 to infinity; AUC_{last} = area under the curve from time of dosing to the time of last quantifiable concentration; C_{max} = maximal plasma concentration; CV% = coefficient of variation percentage; geo. = geometric; hr, hour; IC_{80} = 80% inhibitory concentration; QD = once daily; $t_{1/2}$ = half-life; SD = standard deviation.

Supplementary Table 2. Baseline predictors for grade 3+ treatment-related anemia based on Cox regression models

**Predictor**	**Adjusted HR (95% CI)**	**Adjusted *P*-value**
Baseline hemoglobin, g/dL	0.55 (0.41 to 0.73)	< .001
Dose (120 3/4 vs 160 3/4)	0.35 (0.14 to 0.86)	.02
Dose (160 3/4, 2/1w vs 160 3/4)	0.31 (0.12 to 0.84)	.02
Number of regimens (more than 3 vs 3 or less)	2.32 (1.17 to 4.58)	.02
Baseline ECOG score (1 vs 0)	1.85 (0.95 to 3.61)	.07

2/1w = 2 weeks on, 1 week off; 120 3/4 =120 mg QD 3 days on, 4 days off; 160 3/4 = 160 mg QD 3 days on, 4 days off; CI = confidence interval; ECOG = Eastern Cooperative Group; HR = hazard ratio; QD = once daily.

Supplementary Table 3. Patients with a response to camonsertib across the 3 dose groups

Dose level	Tumor type	Genotype	Alteration	Allelic status	Other genomics	Response	Lines of prior treatment	Time on treatment (weeks)	Max reduction from baseline (%)
120 3/4	Ovary	gRAD51C	p.R237*	Biallelic	HRD+	CA-125	5	67	12.5
	Melanoma	BRCA2	p.R2842C	Monoallelic	TMB-H (UV), ALT+, HRD–	cPR	3	94	74
160 3/4	Ovary	gBRCA1	p.E143*	Biallelic/reversion	HRD+	cPR	5	48	49.3
	Breast	BRCA1	pD825fs*4	Biallelic		uPR	7	18	30.4
	RCC	SETD2	p.565*	Indeterminate		uPR	2	17	36.8
	RCC	RAD51C	p.M10fs*25	Indeterminate		uPR	4	22	63.6
	Pancreatic	gATM	p.Y264fs*12	Indeterminate		cPR	1	41	42.9
	Stomach	gATM	p.H2554fs* 10	Indeterminate		cPR	2	45+	34.9
160 3/4, 2/1w	Ovary	gRAD51C	p.R258H	Biallelic		cCR	2	117+	100
	Prostate	gATM	p.R2993*	Indeterminate		PSA	3	112+	N/A
	HNSCC	BRCA1	p.Q54*	Monoallelic	TMB-H (APOBEC)	cPR	1	26	36.7
	NSCLC	gATM	p.R2598*	Biallelic		cPR	4	98+	46.5
	Ovary	SETD2	p.Y2088*	Indeterminate		cPR	4	35	70

2/1w = 2 weeks on, 1 week off; 120 3/4 =120 mg QD 3 days on, 4 days off; 160 3/4 = 160 mg QD 3 days on, 4 days off; ALT, alternative lengthening of telomeres; APOBEC = apolipoprotein B mRNA editing enzyme catalytic polypeptide; CA-125 = cancer antigen 125; cPR = confirmed partial response; CR = complete response; HNSCC = head and

neck squamous cell carcinoma; HRD, homologous recombination deficiency; max = maximum; N/A = not applicable; NSCLC = non-small cell lung cancer; PSA = prostate-specific antigen; QD = once daily; RCC = renal cell carcinoma; TMB-H = tumor mutation burden-high; uPR = unconfirmed partial response; UV, ultraviolet.+ indicates that treatment was ongoing at time of data cutoff.

Supplementary Table 4. Comparison of key analyses in the initial Phase 1 manuscript vs this updated analysis

Parameter	Initial Phase 1 manuscript[(1)]<br>(N = 120)	Dose optimization analysis<br>(N = 119)
Treatment duration		
Min follow-up[a], weeks	21	45
Range (min, max), weeks	0.4, 65.9	0.4, 117.1
Dose levels tested	All dose levels<br>(included subefficacious and non-tolerated doses)	120 3/4; 160 3/4; 160 3/4, 2/1w dose levels[b]<br>(3 tolerable and efficacious dose levels were compared to determine optimal monotherapy dose)
Safety analyses	TRAE incidence presented by 5/2 vs 3/4 dose schedule<br>Reported based on DLTs and TRAE/TEAEs	TRAE incidence compared across the 3 dose groups<br>Dose reduction and transfusion incidence<br>Timing of onset of grade 3 anemia vs grade 3+ neutropenia or thrombocytopenia
Efficacy analyses		
Summarized by:	Dose: patients dosed at more than 100 mg/day, 100 mg/day or less, and all<br><br>Tumor type or genotype: included patients dosed at more than 100 mg/day	Dose: patients dosed at 120 3/4; 160 3/4; 160 3/4, 2/1w; and all<br><br>Genotype: all efficacy evaluable patients treated at one of the 3 dose levels
Additional responses reported:		Responses in 4 additional patients: *SETD2* RCC, *RAD51C* RCC, g*ATM* pancreatic, and g*ATM* gastric<br><br>The responses in the 2 patients with g*ATM* occurred late, at 30 and 33 weeks, so longer-term follow-up was critical to capture these<br><br>*RAD51B/C* was highlighted as a genotype of interest

2/1w = 2 weeks on, 1 week off; 120 3/4 = 120 mg QD 3 days on, 4 days off; 160 3/4 = 160 mg QD 3 days on, 4 days off; DLT = dose limiting toxicity; max, maximum; min = minimum; RCC = renal cell carcinoma; TEAE = treatment-emergent adverse event; TRAE = treatment-related adverse event.

[(1)]Yap TA, Fontana E, Lee EK, et al. Camonsertib in DNA damage response-deficient advanced solid tumors: phase 1 trial results. *Nat Med.* 2023;29(6):1400-1411.

[a]Time from the last patient being enrolled to the data cutoff date.

[b]See Supplementary Figure 1 for a schematic of all of the monotherapy dose levels and schedules that were evaluated across the study.

Supplementary Figure 1. Comprehensive dose-finding strategy.

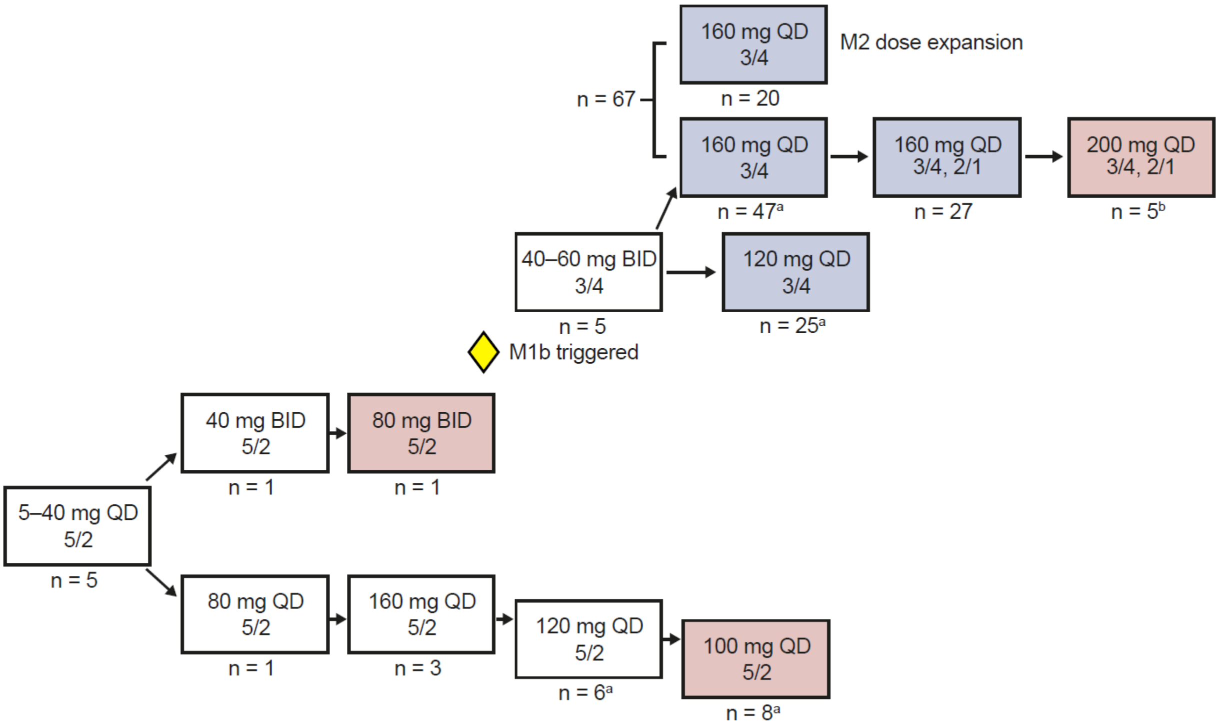

3/4 = 3 days on, 4 days off; 5/2 = 5 days on, 2 days off; BID = twice daily; QD = once daily; M = Module.

Red boxes indicate non-tolerated dose levels. Blue boxes highlight the 3 dose levels evaluated in this analysis.

[a]Includes patients enrolled in the M1c food effect study (single dose in fed state on day –3 and in fasted state on day 1). Patients continued at the following dose levels from day 1: 120 5/2: n = 1; 100 5/2: n = 2; 160 3/4: n = 3; 120 3/4: n = 6.

[b]The last 2 patients were de-escalated in week 1 of treatment based on observed lack of tolerability in 2 of the 3 initial patients (n = 1 had dose-limiting toxicities of grade 4 thrombocytopenia and grade 3 anemia).

Supplementary Figure 2. Pharmacokinetics of camonsertib at **A**) 120 3/4, **B**) 160 3/4, and **C**) 160 3/4, 2/1w dose levels.

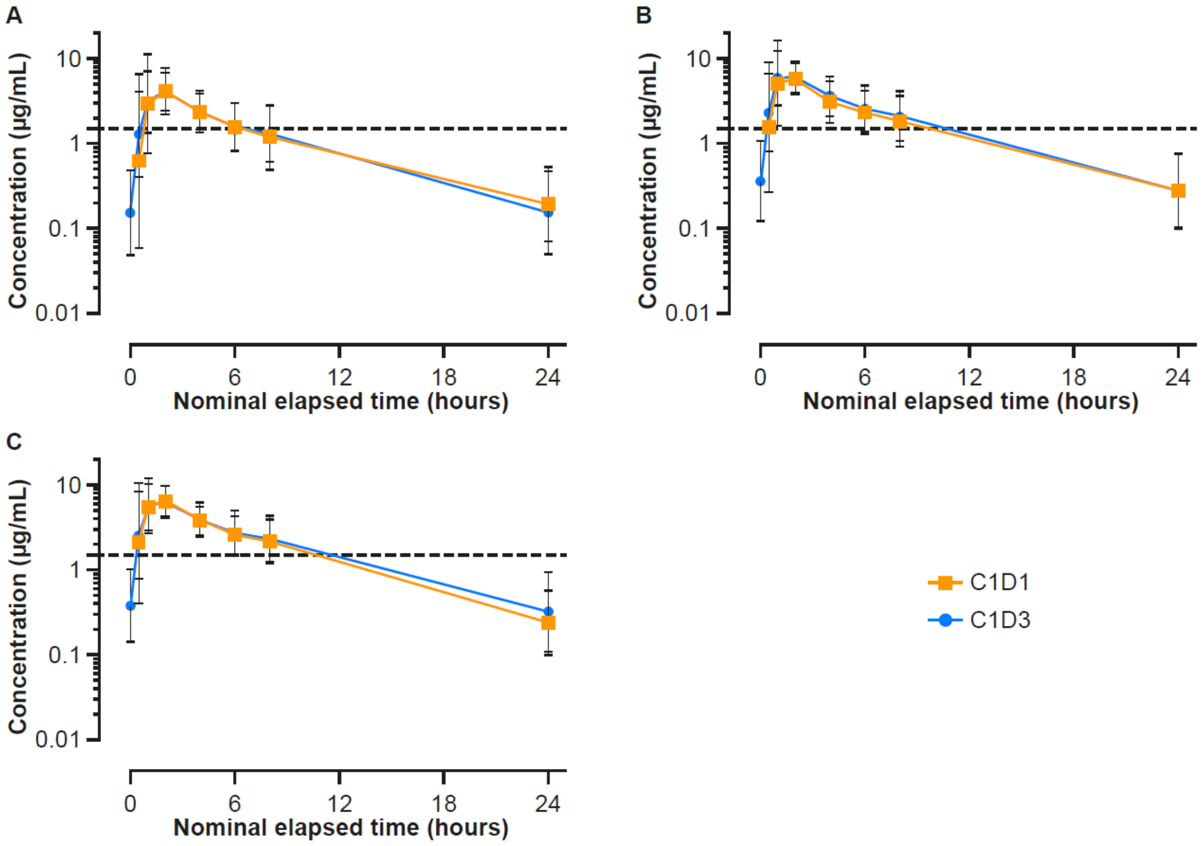

2/1w = 2 weeks on, one week off; 120 3/4 = 120 mg QD 3 days on, 4 days off; 160 3/4 = 160 mg QD 3 days on, 4 days off; C = cycle; D = day; IC_{80}, 80% inhibitory concentration; pCHK1 = phosphorylated checkpoint kinase 1; QD = once daily.

Note: The dashed line represents the pre-clinical xenograft pCHK1 IC_{80} as the target for efficacy.

Supplementary Figure 3. An intermittent weekly dosing schedule enables monocyte and reticulocyte recovery. Changes in monocytes (left axis) and reticulocytes (right axis) over time are shown for patients treated at the 160 3/4, 2/1w schedule. **A)** Patient had a RECIST complete response and remained on the same dose and schedule for 89+ weeks as of the data cutoff. **B)** Patient was on treatment for 35 weeks with no dose changes and best response of confirmed partial response. **C)** Patient was on treatment for 71+ weeks with prolonged stable disease and no dose changes.

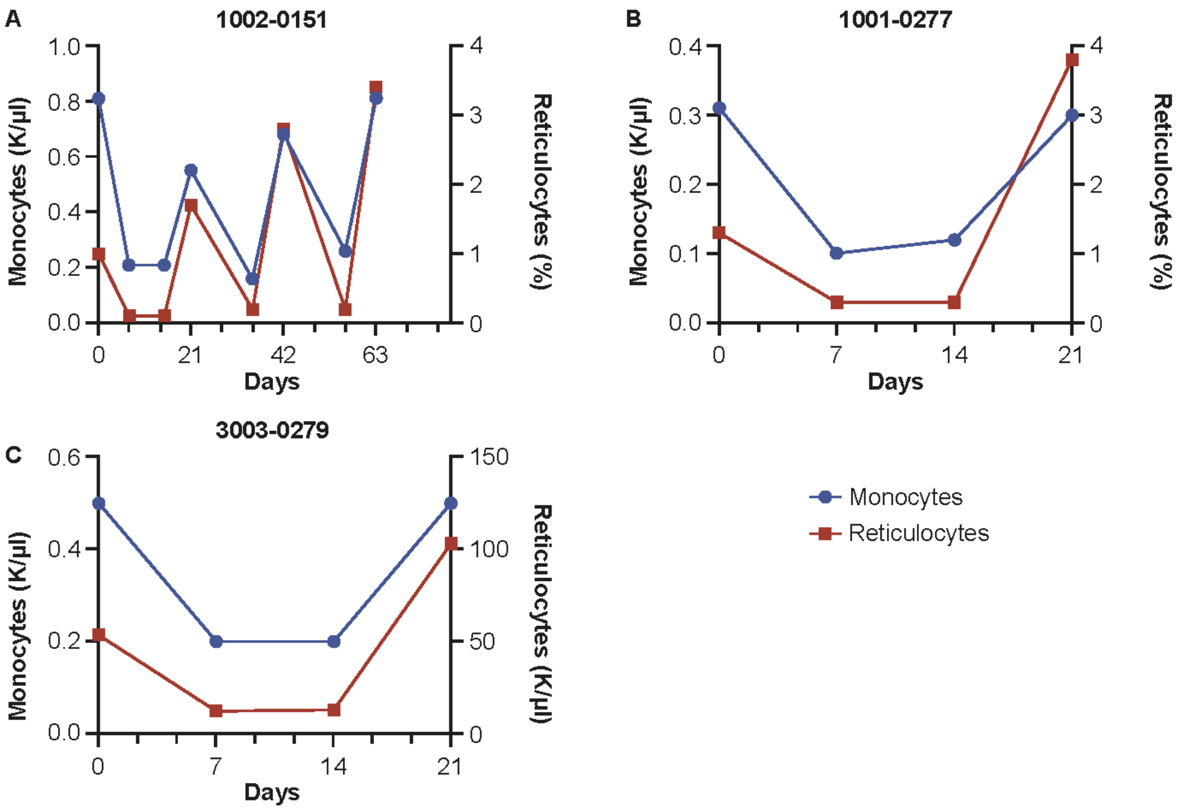

160 3/4, 2/1w = 160 mg QD 3 days on, 4 days off; 2 weeks on, 1 week off; QD = once daily; RECIST = Response Evaluation Criteria in Solid Tumors.

Supplementary Figure 4. Onset of grade 3+ related hematologic events in the first 12 weeks. **A**) Cumulative event rate of grade 3 anemia. **B**) Cumulative event rate of grade 3+ neutropenia and/or thrombocytopenia.

A

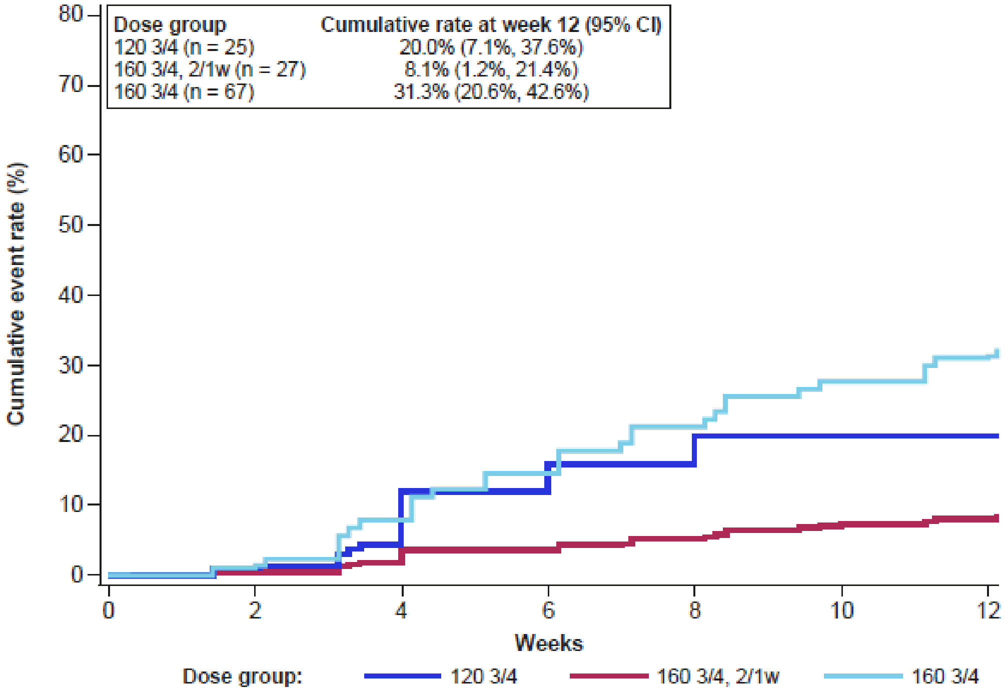

B

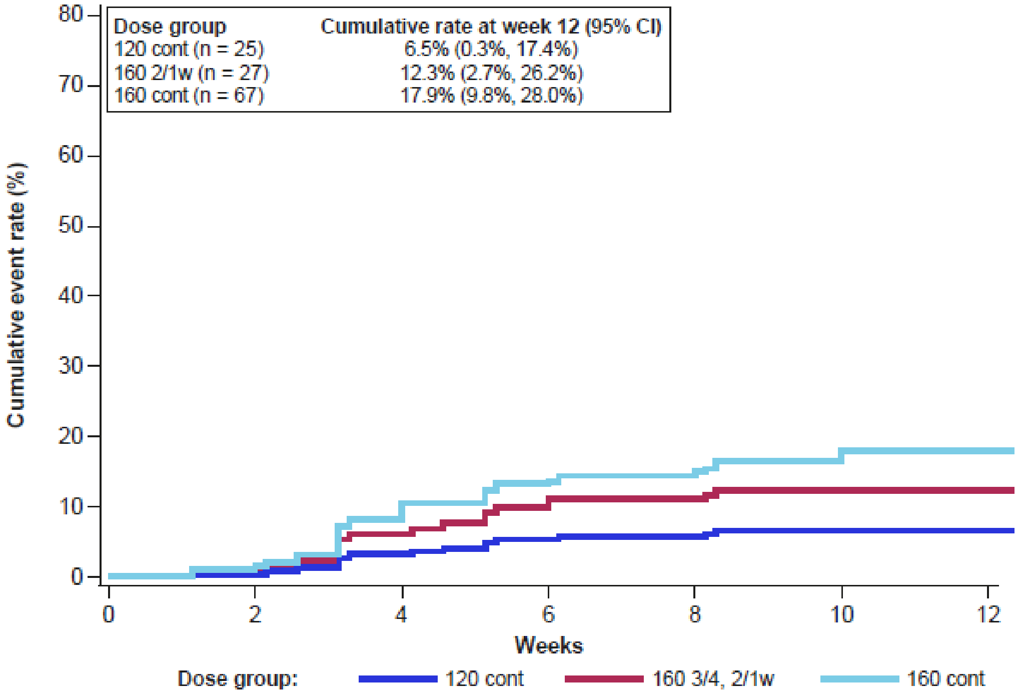

2/1w = 2 weeks on, 1 week off; 120 3/4 = 120 mg QD 3 days on, 4 days off; 160 3/4 = 160 mg QD 3 days on, 4 days off; CI = confidence interval; cont = continuous; QD = once daily.

Supplementary Figure 5. Camonsertib monotherapy dose reductions (for any reason).

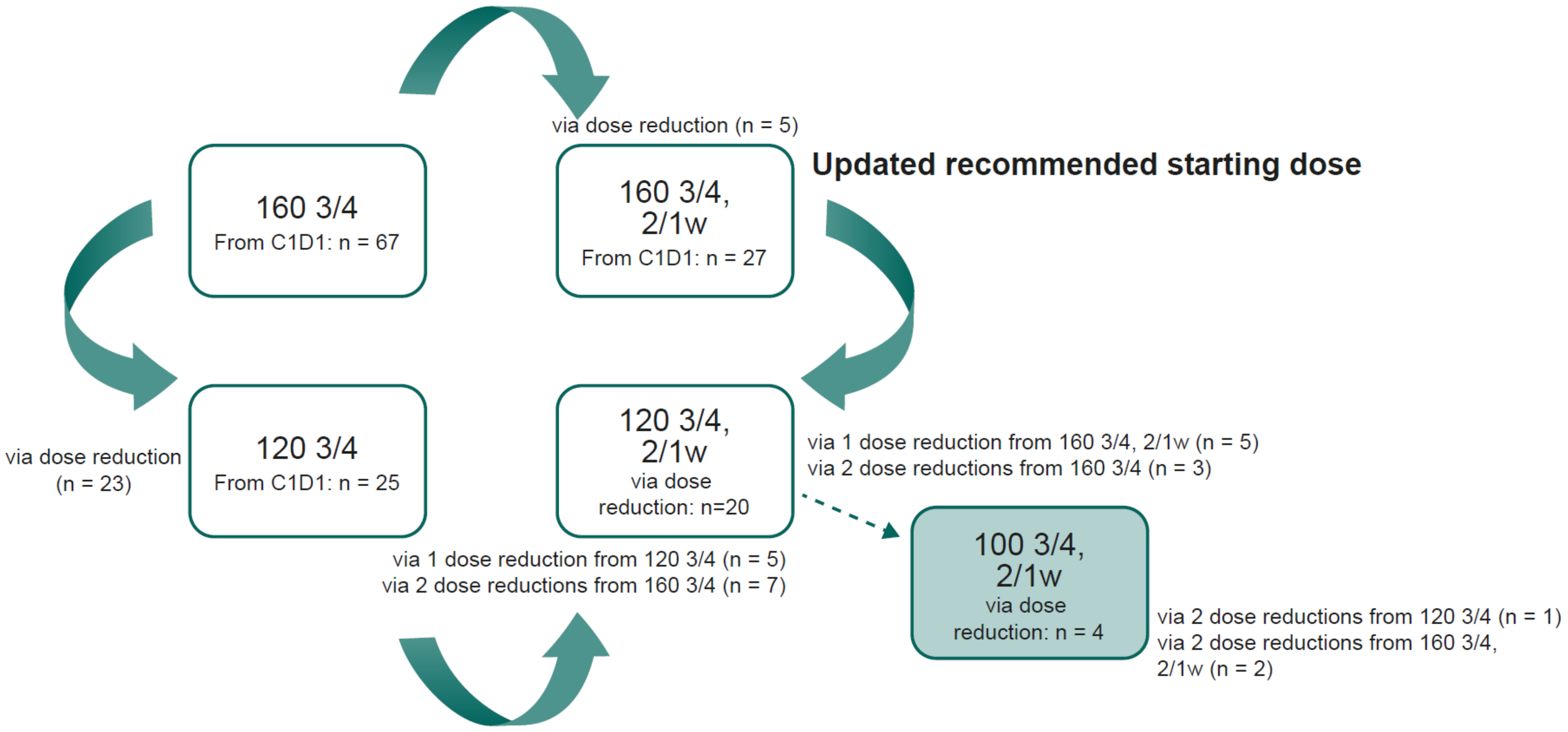

2/1w = 2 weeks on, 1 week off; 120 3/4 = 120 mg QD 3 days on, 4 days off; 160 3/4 = 160 mg QD 3 days on, 4 days off; C = cycle; D = day; QD = once daily.

Supplementary Figure 6. Time on treatment for patients that reduced to 120 3/4, 2/1w.

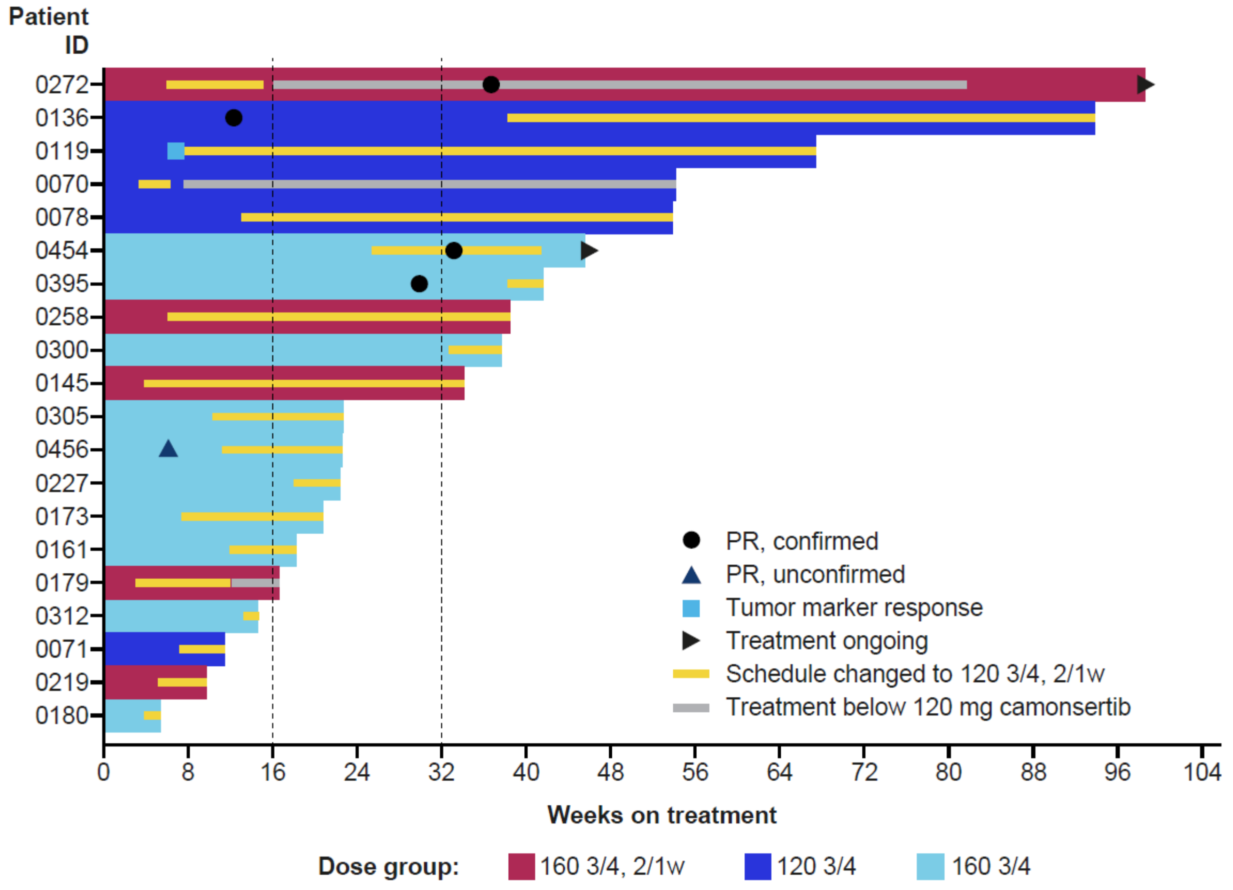

2/1w = 2 weeks on, 1 week off; 120 3/4 = 120 mg QD 3 days on, 4 days off; 160 3/4 = 160 mg QD 3 days on, 4 days off; PR = partial response; QD = once daily; TRAE = treatment-related adverse event.

Note: 1 patient from 160 3/4, 2/1w group had dose reduced to 120 3/4, 2/1w due to a medical history of grade 2 anemia (not a TRAE).

Supplementary Figure 7. Comparison of patient populations in the initial Phase 1 manuscript vs this updated analysis

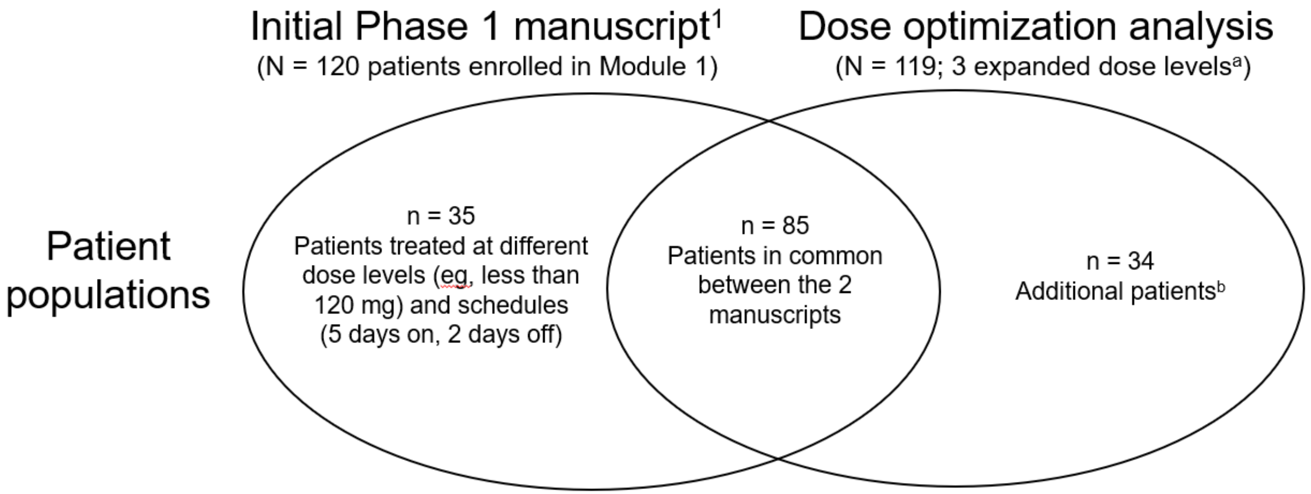

[1] Yap TA, Fontana E, Lee EK, et al. Camonsertib in DNA damage response-deficient advanced solid tumors: phase 1 trial results. *Nat Med*. 2023;29(6):1400-1411.

[a] See Supplementary Figure 1 for a schematic of all monotherapy dose levels and schedules evaluated across the study. The expanded dose levels evaluated in this analysis are denoted.

[b] Includes patients enrolled in Module 1 and in Module 2 biopsy backfills.
